# A Case Report of Methicillin-Resistant Staphylococcus aureus-Related Pericardial Empyema: A Deadly Cardiac Risk

**DOI:** 10.7759/cureus.70684

**Published:** 2024-10-02

**Authors:** Mohammad Hazique, Sara Ahmed, Sehneet Grewal

**Affiliations:** 1 Internal Medicine, Nuvance Health, Poughkeepsie, USA; 2 Internal Medicine, Dow University of Health Sciences, Civil Hospital Karachi, Karachi, PAK

**Keywords:** cardiac tampo, hemorrhagic perica, methicillin-resistant staphylococcus aureus (mrsa), mrsa bacter, pericardial diseases

## Abstract

Pericardial empyema, a rare but often fatal condition, is frequently diagnosed postmortem. Staphylococcus aureus is the most common causative organism. This case involves a 73-year-old male with diabetes and hypothyroidism who presented with chills, fever, and a persistent skin infection. Cultures revealed Methicillin-resistant Staphylococcus aureus (MRSA), and he was treated with intravenous Vancomycin. On the third day, the patient experienced sudden chest pain, and examination revealed muffled heart sounds, jugular venous distension, sinus tachycardia, and diffuse ST elevation. Transthoracic echocardiography (TTE) showed a large pericardial effusion. An emergent pericardiocentesis was performed, followed by a pericardial window, but the patient later developed a clot, leading to the removal of 1000 ml of the bloody fluid. He subsequently became dyspneic and experienced pulseless electrical activity, resulting in death from cardiopulmonary collapse. MRSA was confirmed in the pericardial fluid. This case underscores the critical need for prompt diagnosis and treatment of pericardial empyema due to its high mortality risk and often ambiguous clinical presentation.

## Introduction

Although rare, Methicillin-resistant Staphylococcus aureus (MRSA)-induced pericardial empyema is a severe and life-threatening condition that arises from MRSA infection. Dissemination to the pericardium triggers an immune response. This condition causes the buildup of pus in the pericardium, increasing intrapericardial pressure and impairing cardiac filling [1].

Symptoms include chest pain, fever, shortness of breath, hypotension, and tachycardia. The infection spreads from a primary site, often secondary to pneumonia or chest trauma, or from nearby structures, such as a deep tissue skin infection. Cardiac tamponade can result in decreased cardiac output, hypotension, and, ultimately, cardiogenic shock if not promptly treated. MRSA infections in pericardial empyema cases are notably severe due to their resistance to standard antibiotics. While the prevalence is not explicitly high, the impact of MRSA in these cases is significant, leading to complicated treatment and increased mortality risk. The severity is amplified by complications like cardiac tamponade, which requires immediate intervention [2].

## Case presentation

A 73-year-old male presented with a significant medical history of type 2 diabetes mellitus and hypothyroidism. The patient also had a strong family history of ischemic heart disease and was an ex-smoker and tobacco user. Notably, he was allergic to penicillin and Benadryl. The patient presented with fever and chills, accompanied by a non-healing skin infection on his right arm persisting for two weeks. Upon admission, he was alert and oriented. Vital signs were as follows: blood pressure 120/82 mmHg, pulse 95/min, temperature 99°F, respiratory rate 16/min, and SPO2 95% on room air. A physical examination of the patient revealed a small skin lesion on his right arm along with signs of inflammation and other signs consistent with pericardial effusion, which was initially asymptomatic. The patient reports experiencing shortness of breath more prominently when in a supine position (orthopnea) and retrosternal chest pain. Additional compressive symptoms observed include hoarseness, nausea, dysphagia, and hiccups, suggesting the effusion's impact on nearby structures.

On examination, the apical impulse is either difficult to locate or nonpalpable, indicative of the effusion's effect on cardiac movement. The presence of the Ewart sign is confirmed, characterized by dullness upon percussion at the base of the left lung accompanied by increased vocal fremitus and bronchial breathing sounds. This sign points to the compression of lung parenchyma by the pericardial effusion. Differential diagnosis of cardiac tamponade, MRSA pericarditis, sepsis, constrictive pericarditis, autoimmune disease, aortic dissection, and myocarditis was made.

Initial laboratory tests indicated an elevated white blood cell count of 28,000/ul and a C-reactive protein level of 25 mg/dl. An electrocardiogram displayed a sinus tachycardia rhythm with diffuse ST changes. Subsequent wound and blood cultures identified Methicillin-resistant Staphylococcus aureus (MRSA) sensitive to vancomycin. However, on the third day of admission, he experienced a critical turn of events.

**Figure 1:**
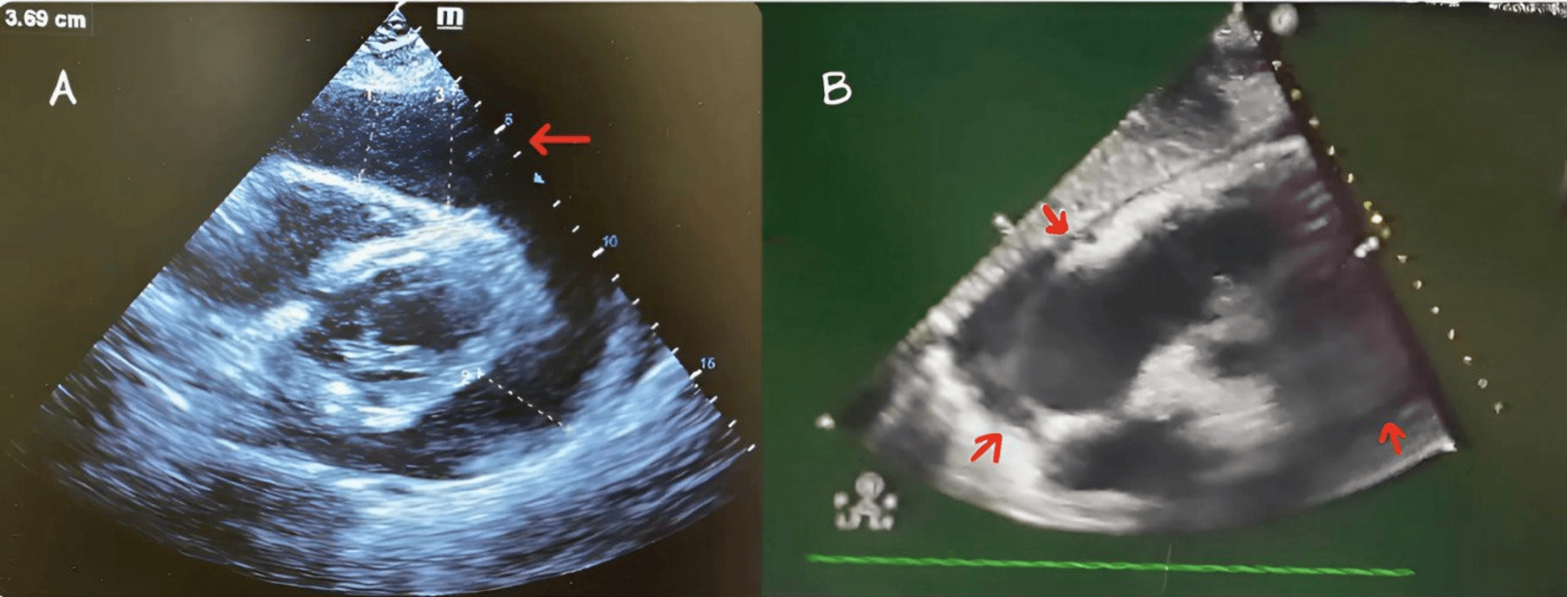
(A) Large pericardial effusion with a maximum diameter of 3.69 cm. (B) Repeat Echo shows no pericardial effusion.

A bedside transthoracic echocardiography (TTE) was promptly conducted, revealing a large pericardial effusion with diastolic collapse of the right ventricle (Figure 1A). These findings were indicative of cardiac tamponade, a life-threatening condition where fluid accumulation in the pericardial sac exerts pressure on the heart, impeding its function.

An emergent pericardiocentesis was performed, yielding 250 ml of cloudy serosanguinous fluid, suggesting an inflammatory or infectious etiology. This procedure was followed by the placement of a pericardial window with a 28 French drain for continuous drainage, which is a standard treatment to prevent re-accumulation of pericardial effusion. The patient was started on intravenous vancomycin. He was placed on continuous telemetry and transferred to the ICU.

The patient’s condition deteriorated the next day with the onset of acute chest pain, accompanied by a significant drop in blood pressure (95/59 mmHg) and an increased heart rate (125 bpm). An electrocardiogram revealed sinus tachycardia with diffuse ST elevation, indicating a possible acute cardiac event. Laboratory findings were remarkable for a marked increase in leukocyte count to 28,000/ul, along with an elevated troponin level of 0.056 ng/ml, suggestive of cardiac injury.

He was found to have a clot formed in the drain, after which 1000 ml of bloody fluid was removed. Although an immediate bedside TTE showed no significant effusion or myocardial rupture (Figure 1B) and intact heart function, the patient rapidly developed dyspnea and progressed to pulseless electrical activity (PEA). Despite aggressive resuscitation measures including chest compressions and epinephrine administration for 30 minutes, the patient did not recover and was declared deceased.

## Discussion

The rapid progression of Methicillin-resistant Staphylococcus aureus (MRSA) in pericardial infections is a critical concern due to its potential for fatal outcomes. The emergence of community-associated MRSA (CA-MRSA) has raised considerable concern, as it is associated with challenging cases of pericardial infections [3]. MRSA infections are associated with greater lengths of stay, higher mortality, and increased costs, emphasizing the urgency of addressing these infections promptly [4]. Several case reports have illustrated the severity of MRSA pericardial infections, such as purulent pericarditis, pericardial abscess, and pericardial tamponade, leading to fatal outcomes [5-6]. These cases underscore the need for immediate medical attention and treatment to mitigate the potentially life-threatening consequences of MRSA pericardial infections. Additionally, the prevalence of MRSA in diabetic patients with tissue infection and the emergence of MRSA infections in communities further emphasize the need for vigilance in recognizing and managing MRSA pericardial infections [7]. Furthermore, the potential complications of MRSA pericardial infections, such as sepsis, cardiac tamponade, and refractory septic shock, highlight the critical nature of these infections and the necessity for comprehensive management strategies [1]. The literature also emphasizes the importance of understanding the epidemiology and prevalence of MRSA infections in different clinical settings to effectively prepare for and address these challenging cases [8].

MRSA infection can lead to pericardial empyema and cardiac tamponade through virulence factors and the host's immune response. The bacteria's ability to resist antibiotics and its production of toxins contribute to the severity of the infection. These factors and the host's inflammatory response can accumulate pus in the pericardial space, leading to pericardial effusion [6]. As the effusion increases, it exerts pressure on the heart, potentially leading to cardiac tamponade, a life-threatening condition requiring immediate intervention. Diagnosing pericardial infections caused by MRSA presents several challenges. MRSA, with its resistance to methicillin and other antibiotics, complicates the treatment of pericardial empyema and raises the risk of progression to conditions like cardiac tamponade [1].

The accurate identification of MRSA in pericardial fluid is crucial for effective management. Advanced microbiological techniques such as culture methods, polymerase chain reaction (PCR) assays, and other molecular diagnostics play a vital role in providing rapid and specific identification of MRSA strains [9]. These techniques enable precise identification of pathogens, including MRSA, in pericardial fluid, thereby aiding in targeted treatment strategies. The diagnostic process typically commences with clinical suspicion based on patient history and symptoms, followed by imaging studies such as echocardiography or computed tomography (CT) scans, which are critical in detecting pericardial effusion and its complications [10]. Echocardiography, in particular, is essential for diagnosing cardiac tamponade, as it enables visualization of fluid accumulation and its hemodynamic effects [10].

The treatment of pericardial effusion often involves antibiotic therapy and drainage of the accumulated fluid. In severe cases, where antibiotic therapy alone is insufficient, surgical intervention becomes necessary. This may include pericardiocentesis, surgical drainage, or a pericardial window to relieve pressure from the accumulating effusion [11]. A surgical pericardial window has the advantage of less re-accumulation of effusion and retreatment. It is important to note that in humans, the treatment of bacterial pericarditis also entails adequate drainage and appropriate antibiotic therapy, and in all cases, there should be a search for the source of the organism infecting the pericardium [12].

Despite several interventions, the patient’s condition deteriorated the next day with a clot formation in the drain, after which 1000 ml of bloody fluid was removed. This large volume suggests a rapid re-accumulation of fluid, potentially due to either the procedural complication or the pathological nature of the effusion. The high viscosity of the pericardial fluid, often seen in purulent pericarditis, might have impeded adequate drainage. In such cases, fibrinolytic therapy with agents like tissue plasminogen activator (tPA) could be considered, but the decision is often complex and based on the clinical scenario [11].

Several factors may contribute to the recurrence of cardiac tamponade. One possibility is that MRSA led to tissue damage during the subacute phase, although the medical management of the infection could also play a role. The most common pathogens in bacterial pericarditis are Staphylococcus aureus (36%), Streptococcus pneumoniae (21%), and Haemophilus influenzae (12%), with the highest mortality rates observed in patients infected with Staphylococcus aureus [12-13].

## Conclusions

As highlighted in this case, it becomes challenging to manage such complex infections needing prompt and appropriate intervention. The outcomes of such cases can vary greatly, with some cases being resolved successfully while others, unfortunately, resulting in fatal outcomes due to the severity of the infection. Pericardiocentesis and pericardiostomy have procedure-related mechanical complications, including cardiac puncture, arrhythmias, pneumothorax, hemothorax, and pneumopericardium.

The potential limitations in the management of this case include delayed diagnosis due to the rarity and complex presentation of the condition, challenges in identifying MRSA as the causative pathogen, and limitations in treatment options due to MRSA's antibiotic resistance. The Infectious Disease Society of America (IDSA) recommends vancomycin or daptomycin for bacteremia and endocarditis, and vancomycin, linezolid, or clindamycin for HA-MRSA or CA-MRSA pneumonia, depending on local resistance profiles of clinical MRSA isolates. However, the appropriate management of suspected or high-risk cases remains unclear.
